# Contribution of the TIME in BCP-ALL: the basis for novel approaches therapeutics

**DOI:** 10.3389/fimmu.2023.1325255

**Published:** 2024-01-17

**Authors:** Nathaly Poveda-Garavito, Alba Lucía Combita

**Affiliations:** ^1^ Grupo de Investigación en Biología del Cáncer, Instituto Nacional de Cancerología (INC), Bogotá, Colombia; ^2^ Grupo de Investigación Traslacional en Oncología, Instituto Nacional de Cancerología (INC), Bogotá, Colombia; ^3^ Departamento de Microbiología, Facultad de Medicina, Universidad Nacional de Colombia, Bogotá, Colombia

**Keywords:** B-cell precursor acute lymphoblastic leukemia (BCP-ALL), immune system, microenvironment, bone marrow, immune avoidance, immunosurveillance

## Abstract

The bone marrow (BM) niche is a microenvironment where both immune and non-immune cells functionally interact with hematopoietic stem cells (HSC) and more differentiated progenitors, contributing to the regulation of hematopoiesis. It is regulated by various signaling molecules such as cytokines, chemokines, and adhesion molecules in its microenvironment. However, despite the strict regulation of BM signals to maintain their steady state, accumulating evidence in B-cell precursor acute lymphoblastic leukemia (BCP-ALL) indicates that leukemic cells can disrupt the physiological hematopoietic niche in the BM, creating a new leukemia-supportive microenvironment. This environment favors immunological evasion mechanisms and the interaction of these cells with the development and progression of BCP-ALL. With a growing understanding of the tumor immune microenvironment (TIME) in the development and progression of BCP-ALL, current strategies focused on “re-editing” TIME to promote antitumor immunity have been developed. In this review, we summarize how TIME cells are disrupted by the presence of leukemic cells, evading immunosurveillance mechanisms in the BCP-ALL model. We also explore the crosstalk between TIME and leukemic cells that leads to treatment resistance, along with the most promising immuno-therapy strategies. Understanding and further research into the role of the BM microenvironment in leukemia progression and relapse are crucial for developing more effective treatments and reducing patient mortality.

## 1 Introduction

**Figure 1:**
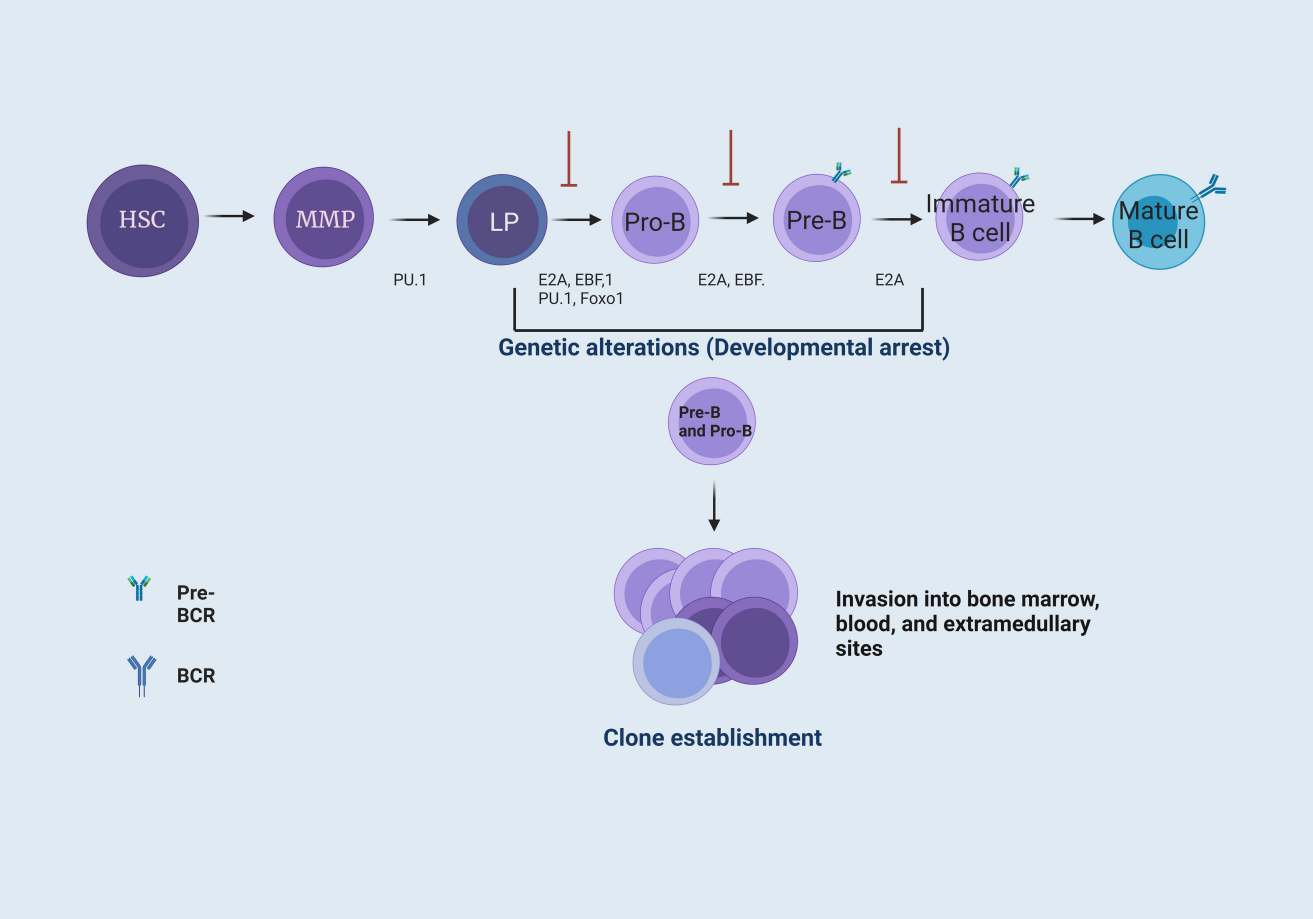
B cell maturation and the establishment of B cell precursor acute lymphoblastic leukemia. B-cell maturation requires different signals coordinated by cytokines, chemokines, and growth factors. These signals are present in the BM microenvironment. In BM, hematopoietic stem cells (HSC) provide the progenitor cells of all blood lineages. This process requires losing their multipotent potential and gaining a specific cell type function (1). The transcription factor PU.1 is necessary for developing lymphoid and myeloid cells. The E2A protein has critical functions in HSC that influence lymphoid progenitor (LP) cells to achieve differentiation towards B cells. These proteins have the function of activating subsequent transcription factors, including EBF1 and Foxo1 (2), which form the key network for B cell lineage commitment. The E2A, EBF1, and Foxo1 network activates Pax5, differentiating progenitor B (Pro-B) cells by repressing genes from other hematopoietic lineages. Subsequently, Pax5, EBF1, and E2A allow differentiation to a precursor B (Pre-B) cell stage. Finally, E2A interactions enable differentiation to an immature B cell stage (3). Genetic alterations, such as point mutations or chromosomal rearrangements at the LP, Pro-B, and Pre-B cell levels, generate a blockage in the differentiation of B cells, where Pro-B cells increase their proliferation and are capable of forming different clones and invade the BM, peripheral blood (PB), and, eventually, extramedullary sites (4, 5).

B-cell precursor acute lymphoblastic leukemia (BCP-ALL) is an aggressive hematologic malignancy involving hematopoietic precursor lymphocytes. This disease is characterized by a block in lymphoid differentiation, resulting in the accumulation of immature progenitor cells not only in the bone marrow (BM) but also in the peripheral blood (PB) and, eventually, in extramedullary sites (**Figure 1**) (6–8). Moreover, within acute lymphoblastic leukemias (ALL), BCP-ALL is the most prevalent in childhood, representing about 85% of the cases, whereas the remaining 15% involve T-lineage ALL (9, 10). Around 60% of BCP-ALL are diagnosed before the age of 20 years old, with a higher incidence in children aged 1-4 years old; it then drops suddenly through childhood (5-14 years), reaching the lowest point between 25 years and 45 years (7, 11). Although the 5-year overall survival (OS) rate was 90% in children with BCP-ALL, only 25% of patients older than 50 years were alive five years after diagnosis (6).

Over the past two decades, there has been a paradigm shift regarding cancer, moving away from viewing it solely as a cellular disease driven by genetic or epigenetic alterations in the genome. Cancer is now recognized as an environmental disease, characterized by active co-evolution among tumor cells and the reprogramming of MSCs, endothelial cells, osteoblasts, adipocytes, and the development of immune evasion mechanisms that constitute the tumor microenvironment (TME) (12). The role of the immune system in tumor initiation and development is highly intricate, with certain immune cells implicated in promoting oncogenesis through an inflammatory process. Concurrently, tumors can instigate an inhibitory tumor immune microenvironment (TIME), suppressing immune function through a complex signaling network, ultimately facilitating the immune escape of tumor cells (13).

In B-cell malignancies, immune dysfunction contributes to relapsed/refractory cases and high mortality. Leukemic cells (LC) proliferate within the BM, a site concurrent with the process of hematopoiesis. Thus, BM is the site of onset, progression, and often recurrence of leukemia. This indicates that LC alter the microenvironment, responding to signals favoring their development at the expense of normal hematopoiesis, including hematopoietic cell inactivity (14, 15). In addition, this provides evidence that intercellular stromal and immune cell crosstalk in the TME allows malignant B cells to evade the host antitumor immune response by enabling immune evasion mechanisms (12).

In BCP-ALL, various soluble immunologic molecules, such as chemokines and cytokines, have been documented. These molecules potentially induce a state of cellular immunosuppression, fostering the survival and proliferation of LC (16, 17). Furthermore this imbalance in the immune system has been implicated in disease progression and drug resistance, ultimately contributing to disease relapse (18).

In the context of BCP-ALL, the contribution of immune cells to the TME is far from being understood; however, it has become a very active area of research in recent decades, with promising results in terms of clinical translational targets to improve disease management. In the present scenario, considering the unsatisfactory outcomes of conventional treatments, alternative approaches encompass immunological therapies. Notably, the use of monoclonal antibodies (mAbs), chimeric antigen receptor T cells (CAR T cells), and natural killer (NK) cells has demonstrated encouraging results (14, 15, 19). There is indication that immune system cells serve as potential biomarkers for prognosis and treatment response (20, 21).

In this review, we delineate the contributions of the TIME that favor immune evasion mechanisms and influence the interaction of these cells in the development and progression of B-cell precursor acute lymphoblastic leukemia (BCP-ALL) (17). Considering the BM as an immunoregulatory organ capable of finely tuning immunity, it emerges as a potential therapeutic target for immunotherapy and immune vaccination (22). Additionally, we emphasize recent therapeutic strategies aimed at re-educating stromal and immune cells within the TME to elicit antitumorigenic effects, including immune checkpoint blockade (ICB) (12). Although the contribution of immune cells to the TME in the context of BCP-ALL is not fully understood, it has become a highly active area of research in recent decades, yielding promising results for clinical translational targets to enhance disease management.

## 2 Bone marrow microenvironment

BM traditionally viewed as a critical hematopoietic organ, supports lifelong normal hematopoiesis and comprises diverse cell types, including hematopoietic stem cells (HSC), osteoblasts, osteoclasts, endothelial, perivascular, mesenchymal stromal, neuronal cells, and pericytes, along with hematopoietic cells (23, 24). These cells physically surround hematopoietic cells, actively regulating hematopoietic processes through the receptors and adhesion molecules (25). Although organized areas of T and B cells are absent in normal BM, it serves as a hub for the functioning, migration, and selective 136 retention of innate and adaptive immune cells (26–29).

**Figure 2:**
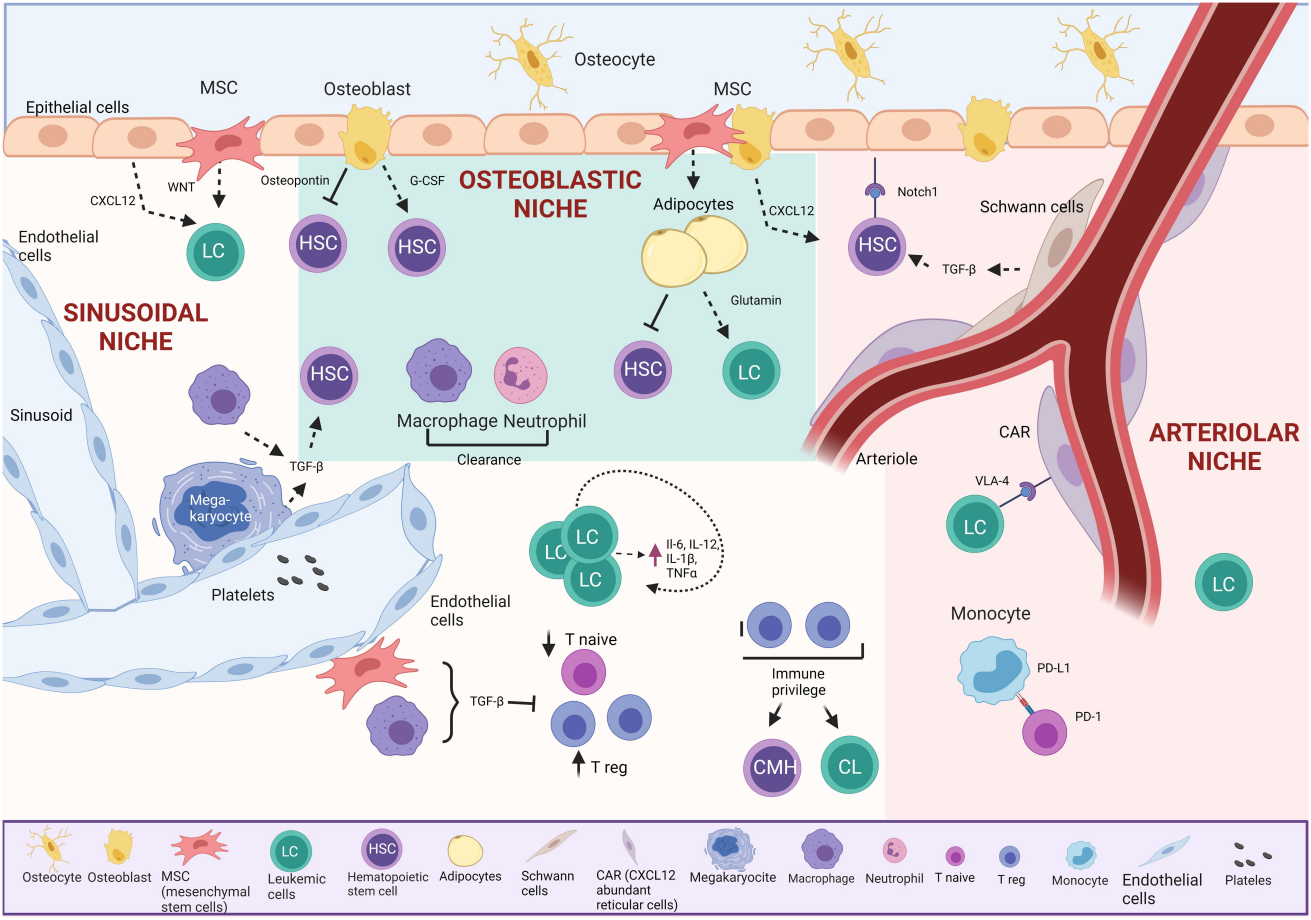
Bone marrow TME in BCP-ALL. The osteoblastic niche maintains HSC in a quiescent state.

Many of the functional properties of HSC and hematopoietic progenitors are determined by their surrounding BM microenvironment, the so-called HSC niche, which provides essential regulatory signals, including soluble cytokines, and cellular interactions for a distinct HSC niches have been recently described: endosteal, arteriolar, and perisinusoidal (30). Each niche has defined characteristics, different cell types, and specific oxygen tensions (31) (**Figure 2**).

The endosteal or “osteoblastic” niche, on the bone’s inner surface, comprises osteoblasts and osteoclasts. Around 20% of HSC are in direct contact with the endosteum. This niche supports HSC through vital cell-cell interactions with osteoblasts, providing essential support, promoting survival, proliferation, and inducing quiescence via secreted signals (27–29, 32, 33). Studies show that osteoblasts play a crucial role in retaining early lymphoid progenitors through the secretion of CXC motif chemokine 12 (CXCL12), while CXCL12 from endothelial cells and mesenchymal stem cells (MSCs) influences HSC maintenance (34).

The arteriolar niche comprises CXCL12-abundant reticular cells (CAR), endothelial cells, nestin-positive MSCs, NG2-positive periarteriolar cells, sympathetic nervous system nerves, and non-myelinating Schwann cells. These cells maintain HSC homeostasis through chemical signaling. Positioned near the endosteum, arterioles in this niche crucially sustain HSC quiescence (35). CAR cells, in direct contact with HSC, are surrounded by endothelial cells in the sinusoids and arterioles (36). Simultaneously, Schwann cells and sympathetic nerves in the arteriole induce HSC quiescence through transforming growth factor-beta (TGF-β) activation, maintaining direct contact with a significant HSC population (34). Additionally, NG2+ periarteriolar cells support HSC quiescence and maintenance in the BM (26, 32, 35, 37, 38).

The sinusoidal niche comprises sinusoidal vessels that are more permeable than arteriolar vessels and are believed to promote HSC activation. Elevated levels of reactive oxygen species (ROS) in HSC induce cell cycle activation, enhancing migration and differentiation capacities (39).

Despite being a rare population, MSCs play a crucial role as key components in the BM. These fibroblast-like cells possess the capability to differentiate into cells of the three mesodermal lineages—osteocytes, adipocytes, and chondrocytes—when exposed to specific stimuli. MSCs consist of various cell subsets, each characterized by distinct localization, expression of specific antigens, and secretion of diverse molecules (40).

Conversely, a pivotal factor in regulating HSC quiescence and metabolism is the signaling associated with BM hypoxia. Contrary to common belief, oxygen tension is not consistent throughout the BM; the endosteal region exhibits the highest pO2, while the perisinusoidal regions are the most hypoxic (41). Another significant factor influencing HSC behavior is BM stiffness. Intriguingly, scaffold stiffness fosters increased HSC adhesion and migration and has been linked to a myelo-erythroid bias ***in vitro***, while softer matrices promote granulocyte differentiation (42).

## 3 Role of immune cells in the constitution of the niche

Besides its role as a hematopoietic organ, BM functions as a primary lymphoid organ, initiating and sustaining immune responses (43, 44). The BM microenvironment hosts various immune cells, including T and B cells, plasma cells, dendritic cells (DCs), neutrophils, and macrophages. These cells constitute an “immune niche” that regulates HSC homeostasis and emergency hematopoiesis through cytokine, hormone, and growth factor secretion, along with the expression of receptors and adhesion molecules (25, 43, 44). This niche appears to safeguard HSC by establishing an immunosuppressive environment or a privileged immunological site, where multiple mechanisms collaborate to prevent immune attacks and even enable prolonged survival of foreign cells (32, 45, 46).

In a healthy individual, approximately 8-20% of BM mononuclear cells are lymphocytes, constituting a T-cell/B-cell ratio of 5:1, distributed in the stroma, parenchyma, and condensed into lymphoid follicles (29). About 1% of this population comprises plasma cells capable of antibody production. CD4+ and CD8+ T cells make up approximately 1.5% and 2.5% of the total BM cellularity, respectively. The CD4/CD8 ratio in the BM is 1:2, reversed compared to peripheral lymph nodes and blood (29). These BM-resident CD4+ and CD8+ T cells exhibit a memory phenotype and secrete cytokines crucial for HSC maintenance (44). In homeostasis, observations in mouse models reveal that CD4+ T cells secrete cytokines such as interleukin-3 (IL-3) and granulocyte-macrophage colony-stimulating factor (GMCSF), modulating normal hematopoiesis (43, 44, 47). Major histocompatibility complex (MHC) class I expression is reported to play a vital role in maintenance and successful long-term reconstitution (48). Experimental approaches demonstrate that activated CD8+ T cells hinder HSC self-renewal capacity and enhance differentiation, while non-activated memory CD8+ T cells support HSC self-renewal, contributing to their maintenance and/or recovery, suggesting a dual role for CD8+ T cells in the BM (49). Memory T cells, both CD4 +and CD8+, are present in the BM, mainly residing in the G0 phase of the cell cycle andinteracting with IL-7-secreting stromal cells to maintain quiescence in the absence of antigen receptor signaling. This underscores the crucial role of the BM in preserving memory T cells (50–52).

About one-third of CD4+ T cells are CD4+CD25+ regulatory T cells (Tregs), co-localizing with hematopoietic stem and progenitor cells (HSPCs) in the endosteum. This may create an immune-privileged BM niche, shielding HSPCs from immune destruction (46). A CD150- expressing Treg subset has been identified, controlling HSC quiescence, pool size, and engraftment through adenosine (53). BM CD169+ macrophages regulate retention gene expression in Nestin+ cells and enhance stromal CXCL12 production, impacting HSC/progenitor retention (54). The BM also houses dendritic cells (DC) and Natural Killer T (NKT) cells, constituting 1-2% and 0.4-4%, respectively (28, 29, 43, 45).

**Figure 3:**
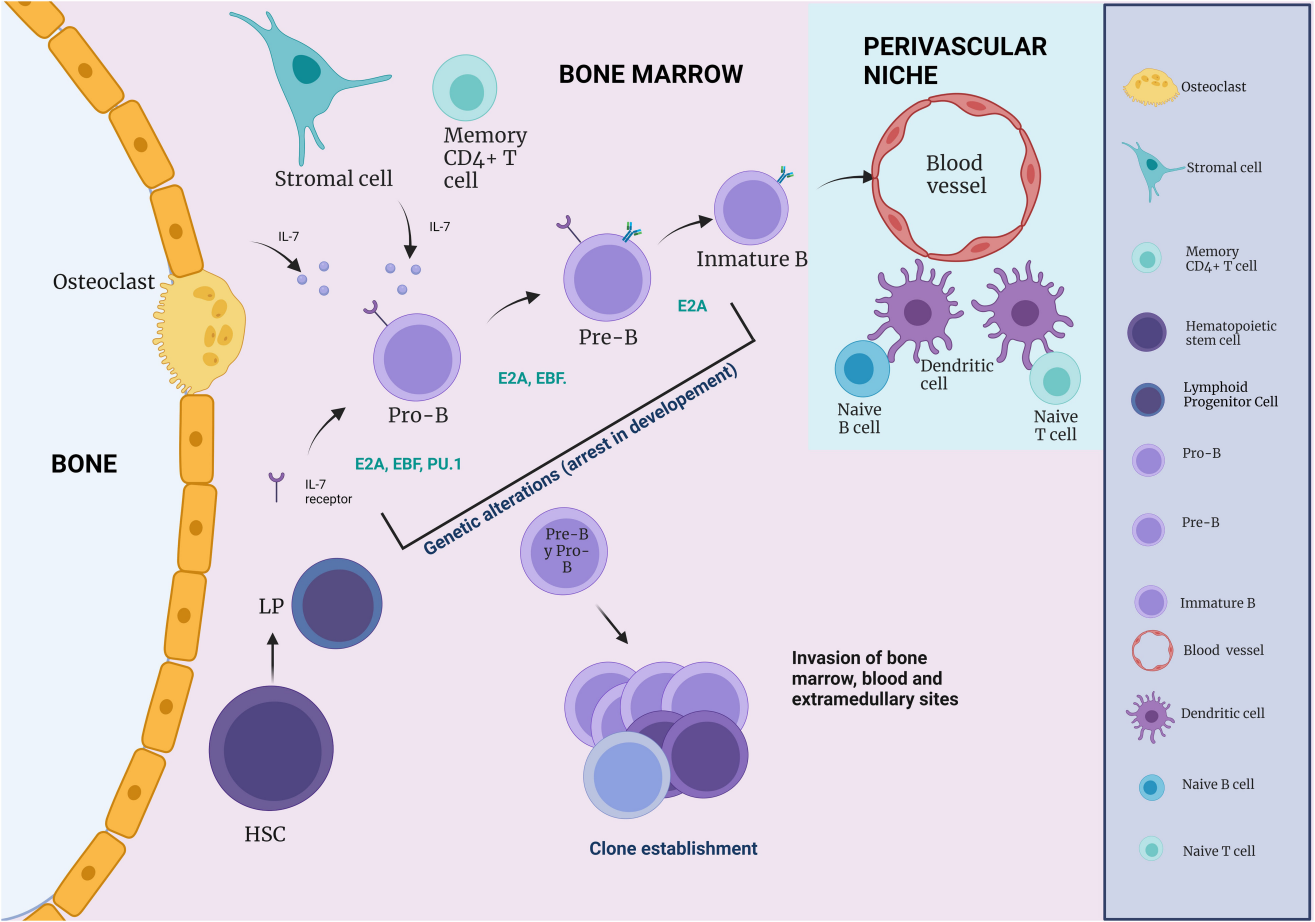
The influence of immune cells on B cell development. During B cell differentiation, HSC and Pre- and Pro-B cells are in close association with CAR cells, whereas pro-B cells are more often in contact with IL-7 secreting stromal cells (IL-7). During B cell development, an immature B cell population can be found in close association with endothelial cells. Naive B and T cells, which can respond to blood-borne pathogens, are within a perivascular niche made up of a network of DCs.

In addition to its role as a primary lymphoid organ by supporting lymphoid development, the BM can act as a host for various mature lymphoid cell types. Thus, the cells of the immune system play a key role in B-cell differentiation (**Figure 3**) (43). The endosteal surface is necessary for the first steps of B cell lymphopoiesis. While osteoblasts, osteoclasts, and CAR cells are required for the earliest developmental stages, IL-7- secreting stromal cells and sinusoidal endothelial cells promote further B-cell maturation (43).

In early lymphoid development, B-cell-potential MPPs remain in the BM, responding to stimuli like IL-7 from osteoblasts. DCs co-localize with naive B cells, secreting survival signals like macrophage migration inhibitory factor (55). Megakaryocytes aid BM maintenance (56, 57). The BM, a repository of mature myeloid elements, mediates myeloid cell delivery to the PB via specific ligands. Naive perisinusoidal B cells can be activated independently by T cell antigens, suggesting perivascular BM immune cells play a vital role in protecting against blood-borne pathogens (58). After antigen exposure, B cells may return to the BM, persisting as plasma cells secreting antibodies. Eosinophils and megakaryocytes contribute to BM maintenance and mobilization through APRIL and IL-6 (56, 57). Neutrophils and monocytes express CXCR4 at steady state, promoting BM retention via CXCL12-induced signaling (59–61).

## 4 Tumor immune microenvironment in BCP-ALL

ALLThe immune surveillance theory suggests that the immune system targets and attempts to eliminate precursor cancer cells (62). Evidence indicates that infiltrating effector T cells can potentially recognize and eliminate leukemic stem cells (LSCs) (63). Cancer cells often employ immunosuppressive mechanisms to hinder the antitumor functions of immune cells, aiding in immune evasion in various malignancies (64). Despite the stringent regulation of BM signals to maintain stability, accumulating evidence suggests that LC disrupt the physiological hematopoietic niche in the BM. This disruption leads to the creation of a leukemia-supportive microenvironment (65–67), where tumor cells modulate immune cells, causing them to lose their ability to recognize the cancer cells as foreign to the host (68–71).

### 4.1 Myeloid-derived suppressor cells

Human myeloid-derived suppressor cells (MDSCs) are characterized as CD33+ and CD11b+. They can be further categorized into granulocytic CD14- and monocytic CD14+ subtypes based on phenotypical and morphological features (72, 73). MDSCs commonly infiltrate both solid and hematologic tumors, exhibiting antitumor activities during early tumor development. Tumor cells and their microenvironment stimulate the proliferation and activation of MDSCs through cytokines and growth factors (74–77). However, as the tumor progresses, signals from the tumor cells and microenvironment can transform the differentiation, maturation, and functions of DCs and macrophages, turning them into immunosuppressive cell types with pro-tumor properties (78). Monocytic MDSCs inhibit T-cell responses in an antigen-unspecific manner, primarily by elevating arginase-1 and iNOS (79). In contrast, granulocytic MDSCs (G-MDSCs) induce immunosuppression mainly through ROS-dependent mechanisms. Patients with BCP-ALL exhibit an increase in G-MDSCs in both PB and BM compared to age-matched healthy controls (80). The immunosuppressive function of BCP-ALL–derived G-MDSCs operates in a ROS-dependent manner and may be associated with STAT3 signaling. This underscores the idea that the accumulation and activation of G-MDSCs represent a novel immune escape mechanism in BCP-ALL patients (80). The clinical relevance of MDSCs is underscored by their close correlation with disease progression and the therapeutic response in clinical settings. Observations in children with BCP-ALL reveal that chemotherapy can impact the numbers of MDSCs and Tregs. Prior to chemotherapy, patients exhibited elevated levels of both MDSCs and Tregs compared to healthy controls. However, during or after chemotherapy induction, there were fluctuations in the numbers of MDSCs and Tregs, showing either an increase or decrease, respectively, compared to pre-chemotherapy levels (81). More recently, BCP-ALL patients displayed a decrease in CD4+ T cell levels alongside an increase in both G-MDSCs and Tregs. The frequencies of G-MDSCs and Tregs directly correlated with the levels of blast cells, CD34+ cells in PB, and BM. This aligns with a prior study indicating that completepostinduction remission is linked to reduced G-MDSCs and Tregs (82). However, chemotherapy-induced myeloid suppressor cells have been observed in some cases. Low doses of cyclophosphamide may enhance the secretion of various inflammatory mediators, including GM-CSF, IL-1 β, IL-5, IL-10, IFN- α, and TNF-α. These factors play a role in the expansion, accumulation, and activation of MDSCs (83), posing a significant hurdle to achieving the desired outcomes. These findings suggest that targeting MDSCs could disrupt immunological tolerance and shift the balance towards sustained antitumor immunity (84).

Monocytic MDSCs suppress T-cell responses in an antigen-unspecific manner, mainly by upregulating arginase-1 and iNOS (79), whereas G-MDSCs induce immunosuppression mainly through ROS-dependent mechanisms. In patients with BCP-ALL, only an increase in G-MDSCs was observed in both PB and BM, compared to healthy controls of the same age (80), and their immunosuppressive function of B-ALL–derived G-MDSCs is a ROS-dependent manner and could be associated with STAT3 signaling. This supports the premise that the accumulation and activation of G-MDSCs is a novel mechanism of immune escape of tumor cells in patients with BCP-ALL (80).

The clinical significance of MDSCs seems to be given by the close correlation between MDSC numbers, disease progression and clinical therapeutic response to therapy. It has been observed that chemotherapy can alter the number of MDSCs and Tregs in children with BCP-ALL. It was found that patients before chemotherapy showed increased numbers of both MDSCs and Tregs as compared to healthy controls. However, during or after the induction of chemotherapy, patients showed increased or decreased numbers of MDSCs and Tregs, respectively, compared to before chemotherapy (81). More recently, a reduction in CD4+ T cell levels and an increase in both G-MDSCs and Tregs were observed in BCP-ALL. In addition, G-MDSCs and Tregs frequencies were directly correlated with the levels of PB and BM blast cells and CD34+ cells. Similarly, in a previous study, complete postinduction remission was associated with reduced G-MDSCs and Tregs (82). Nevertheless, chemotherapy-induced myeloid suppressor cells have been observed in some cases. It is possible that low doses of cyclophosphamide increase the secretion of several inflammatory mediators, such as GM-CSF, IL-1 β, IL-5, IL-10, IFN- α, and TNFα). These factors have been reported to be involved in the expansion, accumulation, and activation of MDSCs (83), which is a critical barrier to achieving this goal. These observations suggest that targeting MDSCs may prevent immunological tolerance and tip the balance toward long-lasting antitumor immunity (84).

### 4.2 Dendritic cells

Evidence indicates that in the presence of soluble factors in BCP-ALL, monocyte differentiated DCs exhibit an atypical phenotype resembling tolerogenic and tumor associated immunosuppressive DCs (74, 85, 86). This phenotype includes low TNF-α/IL-10 expression and elevated levels of TGF-β, IL-6, and IL-1 β, along with chemokines like CCL2, CCL5, and IL-8, and proteins such as COX2, ALDH1A, VEGF, and MMP9. Collectively, these features contribute to impairing the effector immune response, promoting monocyte recruitment, and fostering the development of cells with immunosuppressive activity, thus supporting the growth, survival, and invasiveness of LCs (87). Additionally, various tumor-mediated mechanisms, such as hypoxia, ER stress, and exposure to tumor-derived cytokines and growth factors, have been implicated in generating immunosuppressive and tolerogenic DCs and M2 macrophages (88, 89). Recent findings show that a conditioned medium containing BMP4 from BCP-ALL cells hinders DC and macrophage differentiation from monocytes. Furthermore, BMP4 overexpression in BCP-ALL cells enhances the generation of immunosuppressive DCs, demonstrating increased protumor activity with elevated expression of immunosuppressive and tumor growth factors, including TGF-β, IL-6, IL-1α, IL-8, IDO1, and MMP9 (87).

In BCP-ALL patients, levels of both conventional and plasmacytoid DCs in PB and BM are diminished at diagnosis. These DC levels are linked to disease extent, being lower in patients with unfavorable prognostic features (90–92). Similar studies have demonstrated a significant reduction in both myeloid DCs (mDCs) and plasmacytoid DCs (pDCs) at the diagnosis of BCP-ALL compared to age-matched controls (91, 92). This reduction is distinctive to BCP-ALL, as T-ALL patients exhibit comparable or increased DC numbers. As a prognostic indicator, patients diagnosed with BCP-ALL consistently display a marked reduction in mDCs and pDCs compared to controls (91, 92).

### 4.3 Monocytes and macrophages

Macrophages play a crucial pathological role in malignant tumors, particularly tumor-associated macrophages (TAMs), contributing to the formation of a TME that supports tumor progression. TAMs achieve this by promoting immunosuppression through the release of cytokines like IL-10 and TGF-β (93). Depending on the microenvironment cues, monocytes/macrophages can exhibit diverse phenotypes and functions. While TAM subpopulations with M1-like characteristics exert antitumor effects, those with M2-like characteristics have protumor effects. However, the heterogeneity of TAMs, in terms of activation phenotype and molecular risk, reveals multiple subsets with distinct functions along a continuum between the M1 and M2 extremes (94). Most TAMs are monocyte derived macrophages, closely associated with inflammation, proposed as the seventh hallmark of cancer (95). In the context of BCP-ALL, numerous studies emphasize the perturbation of the monocyte-macrophage compartments as part of the extensive remodeling of the BM niche, playing a critical role in the initiation, progression, survival, and chemoresistance of BCP-ALL (16, 17, 78, 96–98).

Reports suggest that interactions between LCs and leukemia-associated macrophages (LAMs) can reprogram the BM stroma, promoting tumorigenesis in BCP-ALL. The tumor promoting role of human monocytes in BCP-ALL was initially demonstrated in *in vitro* experiments. Co-culture of BCP-ALL cells conditioned monocytes to an inflammatory phenotype characterized by a specific increase in the chemokines CXCL10 and CXCR3, capable of enhancing the migration and invasive properties of LCs (99). Ex vivo-isolated monocytes from BCP-ALL patients exhibited high CXCL10 production compared to monocytes from healthy donors (94). Additionally, the invasion and spread of BCP-ALL cells were mediated by the expression and activity of matrix metalloproteinase 9 (MMP9) (99). Likewise, recent findings demonstrated that BMP4 overexpression in LCs amplifies the generation of M2-like macrophages with protumor characteristics, evidenced by a decreased TNFα/IL-10 expression ratio and increased levels of CCL2 and IL-6, promoting tumor progression (87). Further studies on macrophage recruitment and various polarization mechanisms in the context of BCP-ALL will be crucial to identify potential targetable pathways.

Recently, it has been reported that both T-ALL and BCP-ALL cells can evade immune surveillance by eluding the effector mechanisms of functional macrophages. The overexpression of CD47 on the surface of LCs serves as a “don’t eat me” signal to macrophages, hindering phagocytosis through binding to signal regulatory protein alpha (SIRPα). In preclinical models of acute myeloid leukemia (AML) and myelodysplastic syndrome (MDS), CD47 blockade has demonstrated improvements in antitumor responses (100). Anti-CD47 antibodies have been shown to stimulate antibody-dependent cellular phagocytosis and enhance the priming and memory responses of CD8+ T cells (100).

Magrolimab, targeting CD47 on tumor cells, including macrophage phagocytosis, is currently undergoing evaluation in several early clinical trials for AML treatment (NCT04435691). Results from a recent Phase I study combining Magrolimab with azacitidine showed an overall response rate of 91% and a complete response rate of 42% in patients with MDS (101).

Phenotypic characterization of macrophages in BM biopsy samples from ALL patients revealed a significant increase in CD68+, CD163+, and CD206+ macrophages, particularly in M2 phenotypes, compared to controls (98, 102, 103). Additionally, a comprehensive assessment of BM biopsies from BCP-ALL patients, using multiplex immunohistochemistry, highlighted a specific reduction in M1-like macrophages and an elevated proportion of M2-like macrophages and MDSCs (102, 103). In another study, an upsurge in immunosuppressive CD14+/HLA-DRlow/- monocytes was observed in the BM and PB at the end of induction chemotherapy in patients who later relapsed during BCP-ALL treatment. This alteration may result from the inflammatory response triggered by cytotoxic treatment or the influence of specific drugs used in chemotherapy (104).

Monocytes play crucial roles in regulating cancer development and progression, with distinct subpopulations exerting opposing effects in these processes (105). Recently, it was discovered that the “non-classical” CD14+CD16+ monocyte subset, expressing high levels of CX3CR1, was significantly elevated in the PB of BCP-ALL patients. This elevation correlated with significantly upregulated CX3CL1 in leukemic BM plasma, suggesting an altered migratory pathway that may guide the recruitment of non-classical monocytes into the BM. Additionally, endothelial cells can secrete CX3CL1 in response to proinflammatory cytokines like IL-1β, TNF-α, and IL-6 (98). Interestingly, this CX3CL1/CX3CR1 axis has been identified as a deregulated pathway associated with various tumors, including multiple myeloma (MM) and chronic lymphocytic leukemia (CLL), playing a crucial role in the cross-talk between cancer cells and the TME (106, 107). Similarly, higher CCL2 levels were observed in leukemic BM plasma at baseline and during relapse. Notably, an elevation of the C5a fraction was observed in the BM plasma of BCP-ALL patients, suggesting its potential influence on macrophage recruitment and M2 polarization in BCP-ALL (98). Finally, utilizing single-cell RNA sequencing and cellular indexing of transcriptomes and epitopes (CITE) to analyze BCP-ALL patient samples revealed an augmentation of a non-classical monocyte subpopulation within the myeloid population compartment of leukemic BM at diagnosis and relapse, with inferior relapse free and OS (16, 17) Thus, non-classical monocytes may be generated in response to leukemia-induced tissue inflammation to restore damaged endothelium in a BM infiltrated by LCs.

### 4.4 Natural killer cells

NK cells are integral components of the innate immune system, characterized by their potent intrinsic cytotoxic potential, playing a crucial role in eliminating viral infections and destroying malignant cells, including leukemic blasts. NK cells identify and eliminate malignant cells by detecting the absence of MHC class I molecules on the cell surface (“missing self”) and the *de novo* appearance of molecules on target cells (“induced self”) (108). The capacity of NK cells to kill their targets or secrete cytokines is governed by the balance between activating and inhibitory signals from cell surface receptors, determining the NK cell’s response to either eliminate or tolerate the target cells (109). Inhibitory receptors, including killer cell immunoglobulin-like receptors (KIRs) (KIR2DL and KIR3DL) and the C-type lectin NKG2A, are specific for various human leukocyte antigen (HLA) molecules on target cells, sending signals that suppress activation (110). Other receptors, such as TIGIT, CD96, TIM-3, and PD-1, have been identified as inhibitors of NK cell function. Activating receptors comprise the natural cytotoxicity receptor (NCR) family, signal lymphocyte-activating molecule (SLAM) family, natural killer group (NKG) (NKp46, NKp30, NKp44, and NKG2D), and immunoglobulin (Ig)-like receptors, such as DNAM-1 (CD226) (111–116).

It remains unclear how activating and inhibitory NK cell receptors are downregulated. However, the disturbance of the balance between activation and inhibitory signals may result in abnormal NK cell function, potentially contributing to cancers, including BCP-ALL (117, 118). Loss of NK cell function, either through impaired expression of activating receptors or tumor-driven downregulation of NK cell receptor ligands, serves as a mechanism for tumor immune escape. Cancer cells can impact NK function through various mechanisms, including the modulation of their surface receptors (119), and the release of soluble immunosuppressive factors such as IL-10 or TGF-β (120). Both TGF-β and soluble NKG2D ligands released by cancer cells contribute to decreased NKG2D expression (121). In BCP-ALL patients, higher levels of blast-derived TGF-β1 were identified as a significant mechanism mediating leukemia-induced NK cell dysfunction.

Coculturing ALL blasts with healthy NK cells resulted in an inhibitory phenotype mediated by TGF-β1/SMAD pathway activation, effectively reversed by TGF-β blockade. Similarly, this activation was constitutively present in NK cells at diagnosis and the end of induction compared to healthy controls and BCP-ALL patients during maintenance (122).

AML is often linked to decreased surface levels of DNAM-1, NKp46, NKp30, and/or NKG2D by NK cells; however, variability exists in BCP-ALL patients (123–125). For instance, increased expression of ligands for NK-activating receptors, Nec2, ULBP1, and UBLP3, has been noted on the surface of pediatric LCs compared to adults with ALL, especially without known molecular alterations (126). Specific phenotypic expression patterns are associated with molecularly defined subgroups of ALL patients. In Philadelphia-positive (Ph+) ALL patients or the MLL-AF4+ subgroup, elevated intensity of NK cell-activating ligand expression is observed. Additionally, high surface expression of NKG2D and DNAM1 ligands is found on BCR-ABL+ blasts, regardless of patient age (126). Cytotoxic assays using neutralizing antibodies identified the Nec-2/DNAM-1 interaction as the critical pathway in NK cell/ALL blast recognition (126).

Contrastingly, a recent study demonstrated a significant relationship with a reduction in the percentage or density expression of a specific NK cell-activating receptor, including NKp46, NKG2C, NKG2D, DNAM-1, CD69, 2B4, NTBA, and SLAMF7, during childhood at diagnosis. This decrease is not confined to a single receptor type or family of activating receptors; concurrent downregulation of multiple receptor types belonging to distinct families was also observed (127). The potential correlation between the expression intensity of ligands for NK cell-activating receptors and susceptibility to lysis could enhance the identification of patients who may benefit most from NK-based immunotherapy and alloreactive donors in the context of haploidentical hematopoietic stem cell transplantation (HSCT). Therefore, the relationship between ligand expression and lysis susceptibility provides robust biological support for treatment selection in specific cases, paving the way for new therapeutic algorithms in modern ALL management (126). It has been reported that alterations in the expression of NCRs on NK cells and NCR ligands by ALL cells may allow them to escape the innate response. It was observed that MICA/B was not expressed at detectable levels in NK-resistant LC, suggesting that the common resistance of BCP-ALL blasts to NK cell cytotoxicity may not be caused by inhibitory pathways commonly relevant in tumors, including HLA-G expression, lack of CAMs, or perforin resistance, but rather by the lack of NK cell activation by MICA/B (128).

It has been reported that low surface activation receptor expression at diagnosis is correlated with low NK cell activity, poor outcome, and an increased risk of relapse (120, 129). In a recent study, high levels of ULBP-1, a ligand for NKG2D but not CD112 or CD155 (ligands for DNAM-1), were correlated with inferior event-free survival. This suggests that in pediatric acute leukemia, effective NK cell immune surveillance may depend more on NKG2D than on DNAM-1 (120, 129, 130). In the same study, a combination of molecules that interact to regulate NK functions has been associated with higher relapse rates during/after chemotherapy and poor survival in childhood ALL. The decreased expression of HLA-C in LCs of patients with KIR2DL1/HLA-C*04 interactions has been correlated with higher relapse rates and poor patient survival. Furthermore, this interaction is an independent predictive factor and could complement the current risk stratification used in the clinical management of pediatric patients, especially those with high-risk features (130).

Although LC resistance is believed to involve defective engagement of activating NCRs rather than activation of inhibitory receptors on NK cells, a link has been established between variation in KIR gene content and its influence on childhood ALL risk in recent years. KIR genes are highly polymorphic and interact with equally polymorphic HLA class I molecules. HLA-C, a dominant KIR ligand, is associated not only with increased susceptibility to BCP-ALL but also serves as a risk factor for late relapse. In this model, C2 ligands impair NK cell-mediated surveillance of LCs at both the leukemia initiation stage and in remission (128, 131).

Research has shown that not all NK cells are equally cytotoxic against leukemia due to differences in receptor gene levels and surface expression. In a subset of BCP-ALL patients, although downregulation of HLA-C and HLA-E surface expression is observed at the initial diagnosis, mRNA levels for C1 and C2 carrying HLA-C alleles and HLA-E are lower in CD34+ leukemic blasts than in residual non-leukemic B cells. The presence of KIR2DL5A, NKp46, FASL, granzyme-B, and PI-9 was found to be correlated with a favorable prognosis. In patients who achieved complete remission after chemotherapy, a positive correlation with the presence of NK cells was observed (109, 132). The presence of NK cells in the BM at the moment of diagnosis is associated with a good prognosis for treatment compared to patients who do not have them (21).

### 4.5 CD4+ and CD8+ lymphocytes

CD4+ T-helper (Th) cells play a dual role in antitumor immunity and tumor immune evasion (133). These cells can differentiate into various subtypes, such as Th1, Th2, Th9, Th17, and Tregs, depending on the cytokine environment (134). The remaining nonmalignant T cells in the BM of acute leukemia patients may actively respond against LCs, with a higher CD4/CD8 ratio at diagnosis correlating with a favorable BM response at day 15, indicating early treatment response (135).

Moreover, stimulation of T cells with CD40L+BCP-ALL cell-pulsed DCs not only induces potent and specific anti-leukemic cytotoxic effectors but also differentiates specific and functional Th-1 CD4 lymphocytes with a memory phenotype. These effectors are poised to reach leukemia-infiltrated tissues and orchestrate the antitumor immune response (136). Both CD8+ and CD4+ T cell lines exhibit cytotoxicity against NH-1 in an MHC-dependent manner, suggesting common and potent immunogenic epitopes expressed on DCs loaded with apoptotic B-ALL cells capable of inducing ALL-directed CD8- and CD4-T cell-mediated immunity (137).

In pediatric BCP-ALL patients, activated tumor-associated neoepitope-specific CD8+ T cells respond to 86% of tested neoantigens, recognizing 68% of neoepitopes despite low mutational burden. These findings underscore the robust antitumor immune responses in pediatric BCP-ALL, emphasizing the importance of immunodominance in shaping cellular immune responses (138).

Studies on the immune profile in adult precursor B-ALL reveal decreased levels of M1-like macrophages, Granzyme B+CD57+CD8+ cells, and CD27+ T cells, along with increased levels of MDSC and M2-like macrophages. Elevated expression of checkpoint molecules PD1 and CTLA4 is associated with poor survival outcomes (103, 139). Additionally, the proportion of PD1+TIM3+ double-positive CD4+ T exhausted cells is linked to an increased risk of relapse and serves as an independent predictor of poor survival (103). Similarly, high TIM-3 expression in CD4+ cells from BM at the initial diagnosis is associated with relapse (140, 141). Ex vivo expansion of PB- and BM-derived T cells from pediatric leukemia patients after induction chemotherapy reveals a higher percentage of PD1+ T cells in the BM than in the PB (142).

A study assessing CD4+ and CD8+ cell counts in the PB of pediatric BCP-ALL patients before and after chemotherapy induction revealed chemotherapy-induced changes in T cell subset frequencies. CD10+ B cells increased before chemotherapy, correlating with elevated CD4+ and CD8+ T cells. However, induction chemotherapy led to a substantial decrease in CD10+ B-cell LCs, corresponding to reduced CD4+ and CD8+ T cells. Despite this shift, the CD4+/CD8+ ratio in BCP-ALL patients before and after chemotherapy remained comparable to healthy controls (143). Another study exploring the immunomodulatory effects of chemotherapy in pediatric ALL observed a transient decrease in most T lymphocyte subsets during chemotherapy, which was restored by days 78 and 85. However, senescent CD3+CD8+CD57+ lymphocytes did not return to pretreatment levels (144).

γδ+ T cells, another T cell type, exhibited higher CTLA-4 expression, and B7-2 ligand expression on blasts was elevated in high-risk BCP-ALL patients. Increased CTLA-4 expression on γδ+ T cells and PD-L1 on LC were associated with poor relapse-free survival in BCP-ALL (139). Collectively, these findings suggest that higher expression of immune checkpoint molecules, such as CTLA-4 and PD-L1, is linked to a poorer prognosis in BCP-ALL, highlighting a distinct immune cell context in the BCP-ALL BM compared to healthy controls, even in monocytes and macrophages.

### 4.6 Regulatory T lymphocytes (Tregs)

Tregs, characterized by FOXP3 expression, are a CD4+ T cell subset crucial for maintaining tolerance to self-antigens and regulating the immune response against infections and tumors (145). Within the TME, Tregs play a pivotal role in suppressing anti cancer cell immunity, contributing to cancer growth, proliferation, and progression (146–150). Disrupting the CXCL12-CXCR4 or CCL3-CCR1/CCR axis hinders Treg migration into the leukemic microenvironment, delaying leukemia progression in murine models (151).

In BCP-ALL, FOXP3+ Tregs contribute to maintaining B-cell lymphopoiesis by controlling physiologic IL-7 production (152). Analysis of key cytokines affecting Treg cell fate reveals elevated levels of IL-10, IL-6, IL-23, and TNF-α in BCP-ALL patients compared to controls. Conversely, TGF-β and IL-17 levels are reduced, potentially influencing the pathogenesis of BCP-ALL. Tregs exert their immunosuppressive activity through IL-10 release, creating an inefficient power to control inflammation and favoring BCP-ALL progression (134, 153). Notably, an increase in Tregs with CD4+CD25+ has been observed in patients with ALL. Moreover, increased expression of inhibitory molecules, including CTLA-4, GITR, and LAG-3, in Tregs suggests that their overactivation may contribute to immune escape in BCP-ALL (154). Recent studies have linked Tregs in BCP-ALL patients to the expression of the transcription factor Helios in FOXP3+ CD4+ cells (155). TGF-β produced by Tregs may regulate Helios, leading to an increased local expansion of the CD4+ CD25+ Treg cell pool, correlating with their immunosuppressive function. Additionally, Helios expression in Tregs may regulate angiogenesis in the BM niche of BCP-ALL through the VEGFA/VEGFR2 pathway (155). These findings suggest that Helios+ Tregs could be crucial for oncogenesis and angiogenesis in BCP-ALL, and higher Helios expression may indicate a more severe disease state (155). Notably, a *de novo* insertional mutation in FOXC1 has been described, reshaping the immune microenvironment by inducing a Treg/CTL shift, creating a suppressive immune milieu, and promoting ALL progression. This mutation decreases FOXC1 levels through hypermethylation modifications and attenuates HADC1 transcription (156).

Tregs can be considered a prognostic factor in cancer, as several studies have demonstrated an increased frequency of Treg cells in the PB and BM of BCP-ALL patients compared to controls (63, 81, 134, 150, 153, 154, 157). Additionally, a higher CD4/CD8 ratio at diagnosis correlates with a favorable BM response to chemotherapy at day 15, attributed to the involvement of non-Treg CD4+ cells in the early treatment response. However, Tregs themselves do not impact the final prognosis in childhood ALL (63). Conversely, in another study, patients with BCP-ALL exhibited a lower number of CD4 +CD25+ cells but higher levels of FOXP3, IL-10, TGF-β, and CD152/CTLA-4 than healthy subjects. Notably, the suppressive capacity of regulatory cell numbers increased with disease severity (158). In conclusion, manipulating Tregs could be a promising therapeutic avenue to enhance the effectiveness of antitumor chemotherapy. Larger studies are needed to confirm these findings and ascertain their clinical implications (150).

## 5 The BM TME/LC crosstalk: resistance treatment

While significant strides have been achieved in ALL treatment in recent decades, the prognosis for patients with relapsed or refractory ALL remains bleak (159). In B-cell malignancies, there exists a reciprocal exchange of signals between BM and primary cancer cells. This bidirectional process can result in immune response evasion, functional alteration of the TME, unbridled cancer cell proliferation, metastasis to secondary tumors, and the development of drug resistance, safeguarding leukemia cells from the effects of chemotherapy (160). Although the precise mechanistic details of this intricate process are not fully elucidated, it encompasses cell-cell interactions and involves soluble factors such as secreted signaling molecules and microvesicles. Recognizing that the TME plays a pivotal role in promoting cancer survival, there is a growing consensus that targeting the TME itself should be a priority in treatment strategies to overcome potential drug resistance in cancer cells (160). Although rapid advances have been made in the treatment of ALL in recent decades, the prognosis for patients with relapsed or refractory ALL remains poor (159). In B-cell malignancies, the BM and primary cancer cells exchange signals in a bidirectional process. This can lead to evasion of the immune response, alteration of TME function, uncontrolled cancer cell proliferation, metastasis to secondary tumors, and drug resistance that protects the LC from chemotherapy (160).

### 5.1 Cell-to-cell interactions: immune–stromal cells interactions in BCP-ALL

It has been established that MSCs play a crucial role in modulating immune cell proliferation, differentiation, and activity, creating a tumor-permissive microenvironment. Within this microenvironment, LCs receive signals necessary for their survival and proliferation, contributing significantly to disease progression and drug resistance or disease relapse (18). Specifically, MSCs in the BM environment protect LCs by secreting chemokines that guide BCP-ALL migration and adhesion to specific protective microenvironment niche cells in the BM. MSCs have been shown to exert immunosuppressive effects by influencing the activity and functions of various immune cells, including the inhibition of T cell proliferation, blocking DC maturation, regulating NKcells and macrophages, and inducing Tregs through the release of interferon IFN-γ and TNF via soluble factors and cell-to-cell contact mechanisms (161).

Gal-3, a protein with glycoconjugate ligands on the cell surface, is crucial in this context. Extracellular Gal-3 produced by MSCs is essential for steady-state BCP-ALL proliferation and viability, as well as for controlling efficient leukemia migration and adhesion to MSCs.

Notably, the loss of stromal Gal-3 production sensitizes BCP-ALL cells to conventional chemotherapy, highlighting its role in drug resistance (162). Hematopoietic progenitors express integrins, including the α4 chain (CD49d), critical for homeostasis, renewal, and homing of hematopoietic stem and progenitor cells in the BM (163, 164). In BCP-ALL, aberrant expression of the VLA-4 integrin, formed by non-covalent association of α4 with the β1 integrin chain, has been linked to poor clinical outcomes and cell adhesion-mediated drug resistance within the TME. Notably, α4 binding to its ligandVCAM-1 mediates signaling to maintain LC survival in the presence of chemotherapy (32, 165, 166).

Previous research demonstrated that natalizumab (NZM), an anti-integrin α4 monoclonal antibody, induces B-ALL cells to release VCAM-1, sensitizing them to chemotherapy (167). Despite this, there are no clinically approved agents targeting α4 for BCP-ALL treatment. A recent study introduced AVA4746, a non-peptidic small molecule integrin α4 antagonist, as a potential strategy to combat drug-resistant BCP-ALL. AVA4746 demonstrated high affinity for binding to BCP-ALL cells, efficiently blocking ligand binding to VCAM-1. Furthermore, AVA4746 caused functional detachment of primary BCP-ALL cells from VCAM-1. In combination with chemotherapy, AVA4746 prolonged the survival of mice in an *in vivo* xenograft model of BCP-ALL, suggesting its potential as a therapeutic approach for drug-resistant BCP-ALL (168).

Galectins, another family of cell surface proteins binding specifically to β-galactoside epitopes, are critical components in the TME and have prognostic potential in leukemia/lymphoma. Galectin-1 and Galectin-3, members of this family, are implicated in the protective interaction between LCs and stromal cells in ALL, playing a crucial role in disease progression (169–171). They promote migration and adhesion of BCP-ALL cells to protective microenvironment niches of MSCs in the BM and contribute to protecting BCP-ALL cells against conventional chemotherapeutic drugs. Specifically, extracellular Gal-3 produced by MSCs is crucial for maintaining BCP-ALL fitness during chemotherapy. Targeting galectin-1 and galectin-3 has been explored as a potential strategy to sensitize BCP-ALL cells to chemotherapy (172–174).

## 6 Immune BM TME in resistance to treatment: strategies for re-educating the TME in BCP-ALL

With a growing understanding of TIME in the development and progression of BCP-ALL, current strategies focus on “re-editing” TIME to promote antitumor immunity. Since the BM is an immune regulatory organ capable of fine-tuning immunity, it may be a potential therapeutic target for immunotherapy and immune vaccination. Over the last decade, significant advances have been made in developing new targeted therapies for specific ALL subsets in adults in conventional therapy is not effective (29).

**Figure 4:**
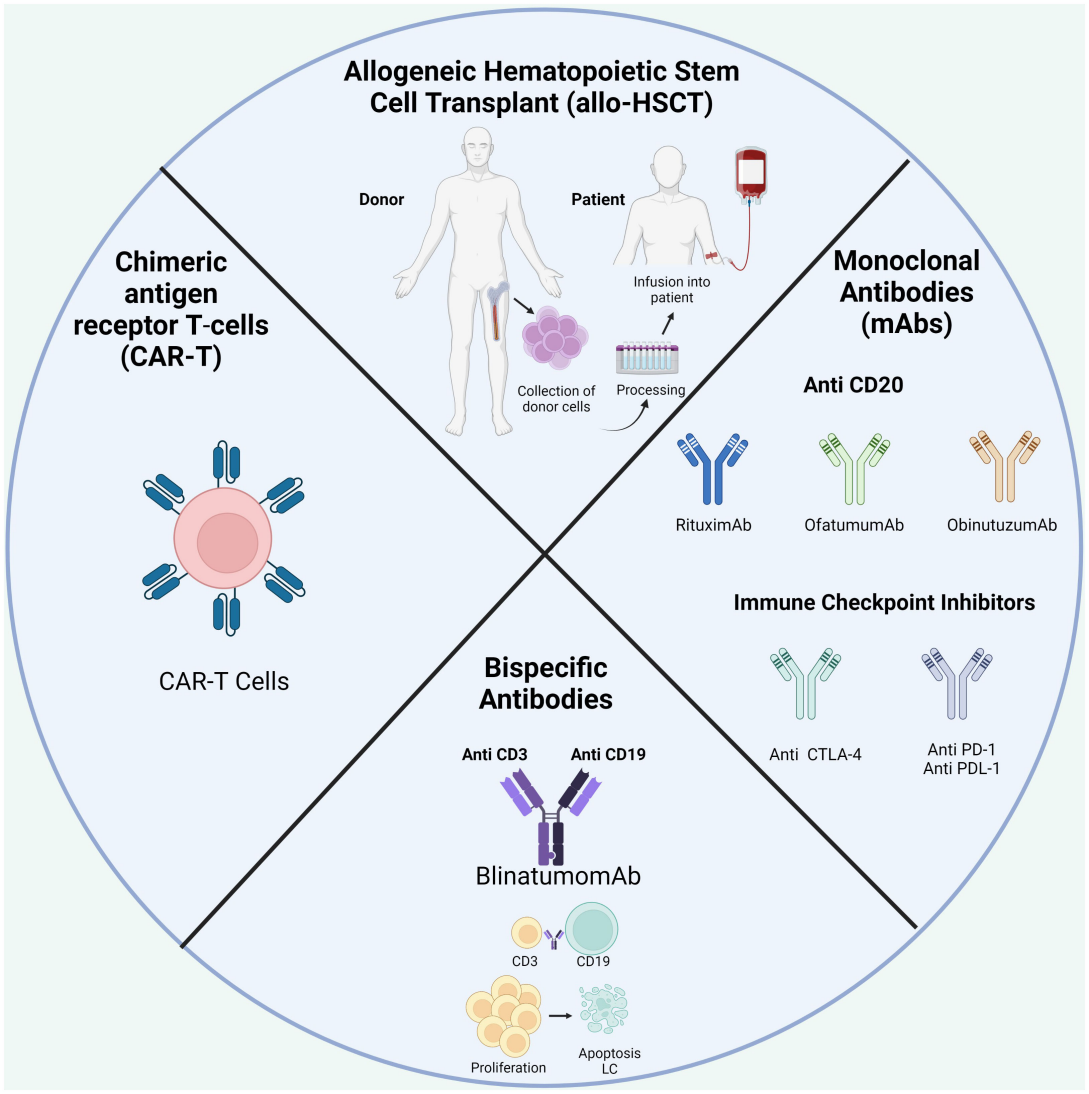
Treatment available in BCP-ALL.

One of the best-known immunotherapies is HSC transplantation (HSCT) after chemotherapy to repopulate the BM niche (**Table 1**; **Figure 4**). The ability of donor immune cells to eliminate host LCs after allo-HSCT is a well-established proof-of-concept; an effective immune response could remove leukemia and is the first example of immunotherapy. Currently, allo-HSCT is seen as a potent immunotherapeutic treatment. Nevertheless, the risk of severe and potentially fatal complications, including graft-versus-host disease, must be considered (19, 175).

**Table 1:** Immunotherapies for BCP-ALL.

Type of immunotherapy	Therapy name	Action mechanisms
HSC transplantation		HSC infusion
Monoclonal antibodies (mAbs)	Blinatumomab	Anti-CD19 and anti-CD3
	Rituxumab	Anti-CD20
	Ofatumumab	Anti-CD20
Antibody-drug conjugate (ADC)	Inotuzumab	Anti-CD20 + calicheamicin
	Ozogamycin	
CAR-T cells	Tisagenlecleucel	CAR-T with CD19
Natural killer (NK) cells	Allogeneically activated NK cells	Activated and expanded NK cells from haploidentical donors
	Autologous NK cells	Enriched and expanded NK cells
	Cord blood NK cells	NK-CAR targets tumor cells

With a growing understanding of TIME in the development and progression of BCP-ALL, current strategies focus on “re-editing” TIME to promote antitumor immunity. Since the BM is an immune regulatory organ capable of fine-tuning immunity, it may be a potential therapeutic target for immunotherapy and immune vaccination. Over the last decade, significant advances have been made in developing new targeted therapies for specific ALL subsets in adults in conventional therapy is not effective (29).

With the introduction of monoclonals antibodies (mAbs) directed at specific antigens on the surface of LCs, such as CD20 and CD19, rituximab, ofatumumab, or obinutuzumab are currently used, which represents a significant advance in the therapeutic of BCP-ALL (14, 22). They work through a number of mechanisms, including antibody-dependent complement-dependent cytotoxicity and direct induction of apoptosis (22).

CD20 expression is recognized in most B-cell malignancies; however, the protein expression level varies in each patient, even within intraclonal subpopulations in an individual patient. It is well-known that CD20 is induced in the context of microenvironment interactions by CXCR4/SDF1 (CXCL12) chemokine signaling, and the molecular function of CD20 has been linked to the signaling propensity of B-cell receptor (BCR) (176). Down-modulation of CD20 is the most basic and frequent cause associated with resistance. Recent reports suggest that genetic and epigenetic mechanisms and transcription factors are correlated with a low CD20 expression in *de novo* tumors and relapsed/refractory disease after using rituximab (177). Moreover, alternative splicing of its 5’ untranslated region controls CD20 mRNA translation and enables resistance to CD20-direct immunotherapies (178), for example, down-modulation of CD20 expression after chemoimmunotherapy with rituximab, resulting in rituximab resistance (179). Other relevant mechanisms for the resistance of CLL patients to rituximab include deficient CDC activity due to increased expression of regulatory proteins, such as CD55, CD59, or factor H, that prevent the formation and deposition of additional C3b and propagation of the complement cascade (180), similarly to polymorphism in the complement component C1qA (181).

On July 2017, the FDA approved blinatumomab to treat relapsed/refractory BCP-ALL (r/r ALL). Blinatumomab is a bispecific CD19-directed CD3 T-cell engager able to activate T cells without the need for additional costimulatory signals (182). The results of a multinational clinical trial show that blinatumomab can induce complete hematological remission in 46.6% of r/r ALL patients, resulting in a survival benefit compared to chemotherapy (183). However, despite this promising data, some patients do not respond to blinatumomab treatment. Loss of CD19 and extramedullary relapse have been observed as mechanisms of resistance to this treatment (184); however, other mechanisms of resistance have not been reported so far. An increased proportion of Tregs was observed in non-responders, which were found to impair T cell response in a contact-dependent manner (20). Exhaustion markers, including PD-1 and CTLA-4, were upregulated by T cells following treatment, in addition to the upregulation of their respective ligands PDL-1 and CD86 at the surface of LCs (185). Equally, blinatumomab-induced inhibitory interactions between T cells and their counterparts expressed on target cells, such as PD-1-PD-L signaling or loss of co-stimulation through CD80 or CD86, might contribute to *in vivo* resistance to therapy (186). Clinical trials with combined treatment approaches with checkpoint-blocking antibodies are currently underway and could be a promising therapeutic strategy in pediatric r/r BCP-ALL to increase antitumor T cell activity (187).

During the last decade, immune-based approaches to target r/r cases of BCP-ALL have emerged based on promising clinical results. Thus, CAR-T cells were approved in 2017 by the FDA for managing refractory or second/later relapsed ALL (188). CD19 CAR-T cell therapy for BCP-ALL has achieved great efficacy with complete remission of 70-90% (189–191). Early trials in BCP-ALL therapy utilized “second-generation” CAR constructs, whereby autologous T cells were transduced with lentiviral vectors expressing a CAR consisting of a single-chain fragment (scFv) from a murine monoclonal antibody specific for CD19, a CD3-zeta domain for T-cell activation, and either a 4-1BB (CD137) or CD28 domain for TCR co-stimulation (192), when expressed in autologous T cells (or donor T cells in the post-transplant context). Upon patient infusion, CAR-T cells engage with antigen-expressing tumor cells in an HLA-independent manner to elicit a cytotoxic response (193). This mechanism has also been used in NK cells designed to express a CAR, making them candidate factors for cancer treatment (194). This approach is effective, especially since many patients have been highly pretreated with chemotherapy. Nevertheless, despite dramatic results seen with CAR-T cell therapy, a significant subset of patients develop resistance.

## 7 Discussion

In relapse, TME is a major contributing factor and controls the migration, survival, proliferation, and, somehow, response to drug treatment in BCP-ALL cells. In the last years, TIME has gained relevance because, according to the soil and seed theory, cancer cells grow in microenvironments that allow it. Thus, the constitution of the immune system could indicate why some therapy in BCP-ALL is not effective; evidence also shows that the immune state of cancer patients does not function in the same way as the immune system of a healthy individual.

New technologies have allowed a better comprehension of TIME in BCP-ALL at the moment of diagnosis, remission, and relapse. Establishing distinct types of TIME in patients with BCP-ALL will aid immunotherapy efforts as a type of treatment in relapsed BCP-ALL. On the other hand, it is important to identify prognostic biomarkers that can be easily sampled through BM or PB, as they provide relevant clinical information to help guide treatment decisions. Moreover, assessing immune landscape changes before and after therapy could improve immunotherapy efforts by informing the context in which therapeutic interventions will be introduced as new therapies are discovered. Similarly, it is necessary to study the periphery immune response such as lymph nodes and PB.

## Author contributions

NP-G: Conceptualization, Investigation, Methodology, Resources, Visualization, Writing – original draft, Writing – review & editing. AC: Conceptualization, Data curation, Formal Analysis, Funding acquisition, Investigation, Methodology, Resources, Supervision, Validation, Writing – original draft, Writing – review & editing.
